# Effectiveness of bent leg raise technique and neurodynamics in patients with radiating low back pain

**DOI:** 10.12669/pjms.38.1.4010

**Published:** 2022

**Authors:** Muhammad Adnan, Aatik Arsh, Babar Ali, Shakeel Ahmad

**Affiliations:** 1 Muhammad Adnan, DPT, MSPT. Coordinator/Lecturer, Department of Health Sciences, City University, Peshawar, Pakistan; 2 Aatik Arsh, DPT, MSPT. Assistant Professor, Institute of Physical Medicine and Rehabilitation, Khyber Medical University, Peshawar, Pakistan; 3 Babar Ali, DPT. Coordinator/Lecturer, Department of Physical Therapy, Abasyn University Peshawar, Pakistan; 4 Shakeel Ahmad, DPT, MSPT. Department of Physical Therapy, Jouf University Sakaka, Saudi Arabia

**Keywords:** Back, Mulligan technique, Neural mobilization, Pain, Physical Therapy

## Abstract

**Objective:**

To compare the effectiveness of bent leg raise technique and neurodynamics in patients with low back pain that radiates up to the knee.

**Methods:**

The pre-test post-test control group study was conducted at Department of Physical therapy, Maqsood Medical Complex and General Hospital Peshawar from February to July 2019. Patients with radiating low back pain of both genders aged 18-60 years were included in the study. Patients were divided into Group-A and Group-B. Group-A patients received Mulligan bent leg raise technique while Group-B patients received neurodynamics. Both groups received five sessions per week, for four weeks. Numeric pain rating scale, Oswestry disability index and goniometer was used to assess pain, functional disability and straight leg raise range before and after the interventions. Data was analyzed using SPSS version 20.

**Results:**

Thirty-two participants with mean age of 38.81±9.94 years, participated in the study. There were no significant differences (P-value>0.05) between the two groups at baseline. Post-treatment, within Group-Analysis showed that all three variables (pain, functional disability and straight leg raise range) significantly (P<0.05) improved in both groups. However, post treatment between Group-Analysis showed that there were no significant differences (P>0.05) between the two groups.

**Conclusion:**

Both neurodynamics and bent leg raise technique significantly improved pain, functional disability and straight leg raise range in patients with low back pain that radiates up to the knee. However, there were no significant differences between the groups who received either neurodynamics or bent leg raise technique.

## INTRODUCTION

Low Back Pain (LBP) is one of the common musculoskeletal disorders which is associated with incredible costs and human sufferings.^1^,^2^ The severity of the problem is obvious from the fact that more than half of the world population, at least once experienced LBP during their lives.^3^ LBP has detrimental effects on physical, psychological and social well-being of the individuals.^4^ The Global burden of diseases study conducted in 2010 reported that LBP is one of the major cause of years lived with disability.^5^ The devastating effects of LBP have been extensively described in the literature.^6^

In majority of LBP patients, pain is experienced in the lumbar and sacral region; however, pain can radiate to lower limbs if there is nerve root involvement.^7^ Different invasive and non-invasive techniques can be used for the management of radiating LBP. Literature suggests that physical therapy is one of the most effective methods of managing patients with radiating LBP.^8^ Apart from electrotherapeutic modalities and exercises, neurodynamics are commonly applied by physical therapists to manage these patients.^9^ Neurodynamics is a nerve mobilization technique which can be applied in different musculoskeletal disorders with neural involvement such as radiating LBP, radiating neck pain and carpal tunnel syndrome.^10^ Previous studies report that neurodynamics is an efficient technique for managing patients with radiating LBP.^11^

As compared to other physical therapy techniques, Mulligan school of thought is a relatively new approach in the field of physical therapy. There is growing evidence to support use of Mulligan techniques in the management of various musculoskeletal disorders.^12^ Mulligan bent leg raise technique has received robust attention from clinicians in the management of LBP that radiates up to the knee.^13^ Despite the fact that physical therapists commonly applied Mulligan bent leg raise technique to patients with radiating LBP, however there is scarce literature available regarding its effectiveness in the management of patients with radiating LBP. Therefore, current study was designed to determine the effectiveness of bent leg raise technique and neurodynamics in patients with LBP that radiates up to the knee.

## METHODS

The pre-test post-test control group study was conducted at Department of Physical Therapy, Maqsood Medical Complex (MMC) and General Hospital Peshawar from February to July 2019. Ethical approval was obtained from Research Ethical Committee of Riphah College of Rehabilitation Sciences of Riphah International University, Islamabad; (Ref: RIPHAH/RCRS/REC/Letter-00344, dated March 21, 2018). Diagnosed patients with radiating LBP (up to knee) having age 18 to 60 years, of either gender was included in the study. Only those patients were included who had unilateral radiation of pain. The diagnosis of the patients was confirmed after consultation with orthopaedic or neurosurgeon. Patients having spinal fracture or major trauma, acute disc bulge, lumbar instability, bilateral radiating pain, sensory and motor deficits, articular pathology, scoliosis, rheumatoid arthritis and other systemic diseases were excluded from the study. After approval from institutional ethical committee, 32 participants with LBP were included who fulfil inclusion criteria and were divided into two equal groups (Group-A and Group-B) through sealed envelope method. The informed consent was taken from the patients.

Group-A was treated with Bent Leg Raise technique along with conventional treatment. The therapist was stand at restricted straight leg raise (SLR) area of patient. The patients knee was flexed and were kept over the therapist shoulder. The therapist instructs the patient to push him away with the patient’s leg and then relax. At this point the therapist extended the patient’s bent knee as much as possible in the direction of the patient’s ipsilateral shoulder. Prolonged stretching was given for several seconds (7-10 seconds) and the leg was lowered to the bed. This technique was repeated three times per session every day for 5 days a week for 4 weeks after applying transcuteanous electrical nerve stimulation (TENS) therapy for 30 minutes before each session.

Group-B was treated with neurodynamic along with conventional treatment. The patient was in a supine position. The therapist stands in front of the patient and places one hand beneath the patient’s ankle to keep away pressure from the peripheral nerves and other hand above the knee joint. The knee was in an extended position and the hip was flexed and the leg was brought to the point where the symptoms reproduced. Slow oscillatory movements were provided by the therapist for several seconds (7 to 10 seconds) after that the leg was brought back to a painless position. This method was repeated three times per session every day for five days per week for 4 weeks after applying TENS therapy for 30 minutes before each session.

Numeric pain rating scale (NPRS), Oswestry disability index (ODI), and goniometer was used to assess pain, functional disability and SLR range before and after the interventions. Data was analyzed using SPSS version 20. Normality of the data was checked using Shapiro-Wilk test. Because the data of pain (NPRS) was not normally distributed, that’s why median and interquartile range (IQR) was calculated for pain. On the other hand, data of functional disability (ODI) and SLR range were normally distributed, that’s why mean and standard deviation(SD) were calculated for these variable. On the basis of findings from data normality test, non- parametric test (Wilcoxon test for within Group-And Mann Whiteny U test for between Group-Analysis) were applied for pain (NPRS) while parametric test (Paired sample T-test for within Group-And Independent Sample T test for between Group-Analysis) were applied for functional disability and SLR range. P-value<0.05 was considered significant.

## RESULTS

A total of 32 subjects (10 male & 22 female) with mean age of 38.81±9.9 years participated in the study. There were 16 participants in each group. Mean weight of the participants was 64.28±9.89 kg while mean height was 5.54±0.91 feet. There were no significant differences (P value>0.05) between the two groups at baseline.

Post-treatment within Group-Analysis showed that all three variables (pain, disability and SLR range) significantly (P<0.05) improved in both groups. However, post treatment between Group-Analysis showed that there were no significant differences (P>0.05) between the two groups (Table-I and Table-II).

**Table I:** Pre and post treatment data of pain (NPRS).

Variable	Treatment groups	Pre-treatment Median (IQR)	Post-treatment Median (IQR)	Within Group-Analysis (Pre and post) P-value*	Between Group-Analysis (Pretreatment) P-value**	Between Group-Analysis (Post treatment) P-value**
NPRS (Pain)	Group-A	7.0(1)	2.5(1)	<0.001	0.136	0.87
	Group-B	6.0(2.75)	2.0(1)	<0.001		

IQR: Interquartile Range, NPRS: Numeric Pain Rating Scale,

* Wilcoxon test

** Mann Whitenny U test.

**Table II:** Pre and post treatment data of disability (ODI) and hip straight leg raise ROM.

Variable	Treatment groups	Pre treatment Mean±SD	Post treatment Median Mean±SD	Within Group-Analysis (Pre and Post) P-value*	Between Group-Analysis (Pretreatment) P-value**	Between Group-Analysis (Post treatment) P-value**
ODI	Group-A	41.93±10.26	18.56±4.58	<0.001	0.421	0.103
	Group-B	38.81±11.36	22.56±7.17	<0.001		
ROM	Group-A	69.50±10.44	82.93±6.72	<0.001	0.210	0.413
	Group-B	67.18±10.94	77.68±7.45	<0.001		

ODI: Oswestry Disability Index, ROM: Range of Motion, SD: Standard Deviation,

* Paired sample T test

** Independent Sample T test.

## DISCUSSION

Physical therapy is one of the key non-invasive treatment approaches for patients with radiating LBP.^14^ Because physical therapy addresses the underlying pathology and biomechanical faults, that’s why physical therapy approaches can effectively manage these patients.^15^ Physical therapy modalities such as TENS and short wave diathermy (SWD) can efficiently relive the pain.^16^ Similarly, physical therapy specific exercises such as stretching and strengthening exercises are helpful in managing the muscle imbalances.^17^ Besides these traditional approaches, pathology specific techniques are applied to manage the pain and functional disability associated with the pathology. Neurodynamics and Mulligan bent leg raise technique are such two techniques which can be used to manage patients with radiating LBP.^18^ Current study was designed in order to determine the effectiveness of bent leg raise technique and neurodynamics in patients with LBP that radiates up to the knee.

Neurodynamic is used in the decreasing of pain and in increasing of range of motion due to facilitations of nerve movements, decreasing of nerve tightness, spreading of the noxious fluid, increasing in neural vascularities and enhancement of axoplasmic flow.^19^ Results of current study showed that neurodynamics significantly reduces pain and functional disability and improves SLR range in patients with radiating LBP. In accordance with results of current study, Yamiin et al. reported that sciatic nerve mobilization can significantly reduces pain. The study also showed that mobilization of nerve was really effective in enhancing the mobility of nerve and improving SLR range.^20^ Hanney et al. reported that use of an neural mobilization through oscillating SLR help in increasing pain-free range of hip flexion. 21 Another study conducted by Adel et al. also reported that neural mobilization is helpful in the reduction of disability, improving discomfort, and centralization of symptom.^22^

To improve flexibility by increasing the hamstrings stretching and to increase range of motion, bent leg raise technique is used, which is advocated by Brain Mulligan. This is a cycles, contraction and relaxation offer to the hamstring by toning muscle and is painless method and may be applied to any participant having limited Straight Leg Raise.^23^ Previous studies have reported that bent leg raise technique is effective for managing patients with LBP that radiates up to the knee.^24^ Tambekar et al. reported that bent leg raise technique techniques give significant results by improving pain, SLR and functional disability.^25^ Hall et al. reported that bent leg raise technique can reduce pain and improve ROM in subjects who have LBP with limited Straight Leg Raise.^26^ Results of current study also showed that pain, functional disability and SLR range can be significantly improved in patients who received bent leg raise technique.

There is limited literature available which compared neural mobilization with mulligan bent leg raise technique. Results of current study showed that there were no significant differences between the participants who receive either neural dynamics or bent leg raise technique. This clearly shows that use of either treatment technique can significantly improve pain, functional disability and SLR range.

### Limitations of the study:

Despite the fact that current study was one of the preliminary study, which determine effectiveness of neurodynamics and bent leg raise technique in the management of patients with radiating LBP, however it has some limitations. Firstly, Current study was conducted in clinical settings, so it was difficult to control confounding variables. Secondly, current study failed to report the combined effects of these two interventions. Small sample size is another major limitation of current study.

## CONCLUSION

Both neurodynamics and bent leg raise technique significantly improved pain, functional disability and SLR range in patients with LBP that radiates up to the knee. However, there were no significant differences between the groups who received either neurodynamics or bent leg raise technique.

### Authors` Contribution:

**MA:** Concept and study design, literature search and literature review, acquisition of data, drafting the manuscript, final approval of the version to be published. He is responsible for the accuracy or integrity of the work.

**AA:** Literature search and literature review, drafting the manuscript, analysis & interpretation of data.

**BA:** Literature search and literature review, acquisition of data, critical revision.

**SA:** Concept and study design, literature search and literature review, critical revision.
